# Polymorphisms of the *IL27* gene in a Chinese Han population complicated with pre-eclampsia

**DOI:** 10.1038/srep23029

**Published:** 2016-03-14

**Authors:** Bin Liu, Yuan Li, Yuan Yao, Hua Li, Hongda Liang, Miaomiao Xin, Liqin Wang, Lei Zhao, Jizheng Lin, Shiguo Liu

**Affiliations:** 1 Department of Rheumatology, the Affiliated Hospital of Qingdao University, Qingdao, China; 2 Department of Medical Imageology, Peking Union Medical College Hospital, Peking Union Medical College and Chinese Academy of Medical Sciences, Beijing, China; 3 Department of Medical Imageology, the Affiliated Hospital of Qingdao University, Qingdao, China; 4 Prenatal diagnosis center, the Affiliated Hospital of Qingdao University, Qingdao, China

## Abstract

IL-27 could inhibit the development of Th17 cells, and the Th17/regulatory T-cell imbalance may reverse maternal tolerance in pre-eclampsia (PE). The aim of this study was to investigate the association between genetic polymorphisms in *IL27* with PE. Three SNPs in *IL27* (rs153109, rs17855750, and rs181206) were genotyped in a Chinese Han cohort of 1040 PE patients and 1247 normal pregnant women using the TaqMan allelic discrimination real-time PCR method. The CC genotypic distribution of rs153109 was significantly higher among cases than controls (19.1% versus 13.3%, odds ratio [OR]: 1.54, 95% confidence interval [CI]: 1.23–1.93, *p* < 0.001), and the CT genotype was found to be significantly lower in cases than controls (41.7% versus 49.0%, OR: 0.74, 95% CI: 0.63–0.88, *p* < 0.001), disputing existing reports indicating the allele frequency of rs153109 is not significantly different between PE patients and controls. Additionally, the CC genotype of rs153109 was significantly more prevalent in PE cases than controls using a recessive model (*p* < 0.001). The allelic and genotypic frequencies of rs17855750 and rs181206 were not significantly different between two groups. Our results reveal that *IL27* polymorphisms may be involved in the development of PE in Chinese Han population.

Pre-eclampsia (PE) is a relatively common, systemic pregnancy disorder characterized by the development of concurrent hypertension (>140/90 mmHg) and proteinuria (>300 mg/24 h) at ≥20 weeks of gestation, that may also be associated with a myriad of other symptoms such as edema, headache, blurred vision, irritability, abdominal pain, and thrombocytopenia^1^,^2^. This gestation-specific syndrome affects about 2–8% of all pregnancies, and is a major cause of maternal and neonatal morbidity and mortality worldwide^3^. Although the etiology and pathogenesis of PE have not been clearly identified, key pathogenic factors may include immunologic factors, shallow extravillous trophoblast invasion into the uterine spiral arteries, genetic and environmental factors, and chronic inflammation. Many parts of the inflammatory network are involved, yielding minor systemic changes that have been considered to be part of the physiology of normal pregnancy; however, the systemic inflammatory response in PE is more severe than in normal pregnancy^4^. This excessive systemic inflammatory response of PE results in endothelial dysfunction and associates increased vascular reactivity preceding the onset of symptomatic clinical disease^5^,^6^. Th17 cells, characterized by the production of IL-17, participate in successful pregnancy as well as in the pathogenesis of diseases of pregnancy, such as recurrent spontaneous abortion and PE. Excessive Th17 cell numbers and high levels of IL-17, IL-6, and IL-1β have been identified in PE, and uncontrolled Th17 cells may emerge as important mediators of inflammation and tissue damage^7^.

Interleukin-27 (IL-27) belongs to the IL-12 family of heterodimeric cytokines including IL-12, IL-23, and IL-35, which help the differentiation and maintenance of Th1 subset cells^8^. IL-27 is a key cytokine that plays a unique role in regulating the initial step of Th cell differentiation^8^. Th17 cells and IL-17 cytokines are regulated by a complex immune network. IL-27 suppresses Th17 differentiation and IL-17 production. Yin *et al.* observed that the expression levels of IL-27 and IL-27 receptor *α* were significantly elevated in trophoblastic cells from the placenta of patients with PE compared with control specimens^9^. Furthermore, the evidence from family-based studies suggests that PE has a heritable component, and genetic involvement plays an important role in its development^10^. Several studies have indicated that many candidate cytokine genes, such as *IL10*, *IL6*, *TNF-α*, and *TGF-β1*, are thought to be associated with the susceptibility of PE^11^,^12^,^13^,^14^. Considering that *IL27* polymorphisms might influence the susceptibility, severity, and outcome of PE^15^, the purpose of study here was to investigate the correlation between *IL27* polymorphisms (rs153109, rs17855750, and rs181206) and the clinical implication of PE in a Chinese Han population, to illustrate whether these SNPs were involved in the development of PE.

## Results

### Clinical characteristics of PE and control groups

The case-control study included 1040 PE patients (30.06 ± 5.69 years old) and 1247 controls (30.32 ± 4.10 years old). The distribution of selected epidemiologic and clinical factors in cases and controls were shown in Table 1. The demographics such as age, age of menarche, and triglyceride levels were similar between the cases and controls (all, *p* > 0.05). The PE group had earlier neonatal gestational age, lower birth weight, higher blood pressure, and higher levels of alanine aminotransferase (ALT), aspartate aminotransferase (AST), urea nitrogen, and creatinine (all, *p* < 0.001).

### Genetic analysis

Allelic and genotypic frequencies of the three polymorphisms in *IL27* (rs153109, rs17855750, and rs181206) among cases and controls were depicted in Table 2. The genotypic distributions of these SNPs were within Hardy–Weinberg equilibrium in the control group (for rs153109, *p* = 0.147; rs17855750, *p* = 0.11; and rs181206, *p* = 0.07).

No significant differences were observed among case and control groups in relation to allelic frequency for rs153109 (40.0% vs. 37.8%, *p* > 0.05). Contrary to this, the CC genotype of rs153109 was associated with a higher risk of presenting PE (OR: 1.54, 95% CI: 1.23–1.93, *p* < 0.001), as the prevalence of this genotype was found to be significantly higher among PE cases as compared with controls (19.1% vs. 13.3%, *p* < 0.001). The CT genotype might be a factor protecting from PE (OR: 0.74, 95% CI: 0.63–0.88, *p* < 0.001), as the prevalence of this genotype was detected to be significantly lower in PE cases than controls (41.7% vs. 49.0%, *p* < 0.001). The proportions of the minor allele for rs17855750 and rs181206 were 0.14 and 0.15, respectively, in cases, and 0.15 and 0.14, respectively, in controls. We did not observe significant differences between individual allelic or genotypic frequencies of the studied *IL27* SNPs in these two groups (*p* > 0.05).

In genetic association studies, statistical power to detect disease susceptibility loci depended on the genetic models tested. Therefore, the genotype frequencies were further analyzed by three genetic models: additive, dominant, and recessive. For rs153109, weak association was also found under the recessive model (CC/CT + TT) (OR: 1.54, 95% CI: 1.23–1.93, *p* = 1.63 × 10^−4^), while rs17855750 and rs181206 were not risk factors for PE based on the three genetic models (all, *p* > 0.05) (Table 3).

### Analysis of Genotype-Phenotype Relationship

Analysis of the rs153109 genotypes and phenotypic characteristics among PE patients were depicted in Table 4. Gestational age at diagnosis, neonatal gestational age, and birth weight of offspring showed statistically significant differences among the three genotypes (all, *p* < 0.05). Patients carrying the CT genotype had lower gestational age at diagnosis (35.04 ± 3.66 vs. 35.81 ± 3.48 weeks, *P*_*C*_ = 0.006), neonatal gestational age (35.91 ± 3.19 vs. 36.61 ± 2.93 weeks, *P*_*C*_ = 0.006), and birth weight of offspring (2507 ± 915 vs. 2686 ± 906 g, *P*_*C*_ = 0.024) than ones with the TT genotype. Additionally, the level of serum TC was higher in patients with the CT genotype than ones carrying the TT genotype.

### Linkage disequilibrium (LD) analysis of the SNPs

**Figure 1:**
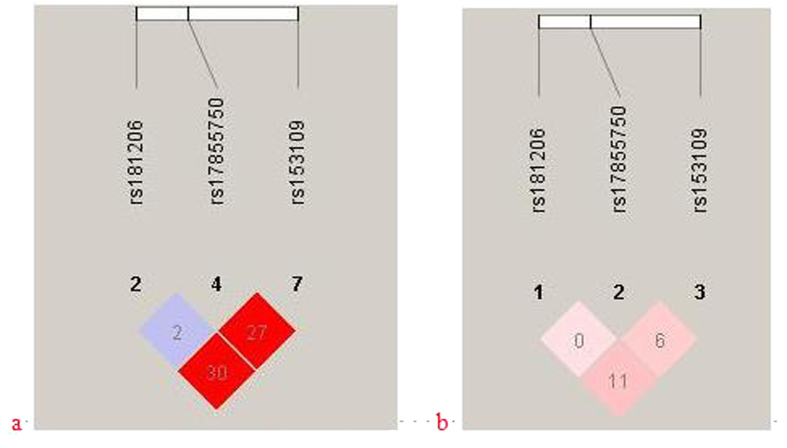
LD analysis of the SNPs in the IL27 gene region. The LD plots were generated by Haploview software v4.2. The number (divided by 100) in the small square represents r^2^ value and ranges from 0 to 1 (the numbers in the figure just shown two digits behind the decimal point). (**a**) The data from HapMap CHB. (**b**) The data analysis between PE patients and healthy controls from our study.

Haploview software was used to further analyze the LD analysis in the *IL27* SNPs. The results from the LD analysis of the SNPs (rs153109, rs17855750, and rs181206) in our study and the data from the HapMap CHB population were shown in Fig. 1. Data from HapMap CHB and the present study illustrated a litter differences.

## Discussion

PE is a common systemic obstetric disorder characterized by the state of excessive inflammatory response. The balance of immunoregulation plays a critical role in the development of PE, which strong activation of the innate immune and a shift towards an inflammatory cytokine profile have been found^16^. Production of Th1 and Th2 cytokines is elevated in PE patients, therefore these cytokines are of interest as possible markers for the development of this disorder^17^,^18^. Significantly higher levels of cytokines such as IL-2, TNF-α, and IFN-γ, but significantly lower levels of IL-4 and IL-10, have been identified in PE patients^19^. Regulatory T (Treg) cells play a crucial role in the development and maintenance of tolerance in peripheral tissues and the induction of transplantation tolerance, as well as in the maintenance of maternal-fetal tolerance^16^,^20^. It has been reported that the decreased number and function of Treg cells may be contributed to induce activate of the inflammatory response characteristic of PE, and increase of Th17 immunity is related to the activation of a Th1 response through modulation of the Th1/Th2 immune balance in PE^19^. Th17/Treg imbalance may reverse maternal tolerance in PE.

IL-27, a complex cytokine with multiple functions, is produced by antigen-presenting cells when they are activated through Toll-like receptor signaling^21^,^22^. IL-27 is a key cytokine that plays a unique role in the regulation of Th differentiation at the initial step, promoting Th1 differentiation and enhancing the activities of Th1 cells^8^. Additionally, IL-27 is also known to act by inhibiting the differentiation of Th2 and Th17 cells^23^,^24^. Th17 cells, characterized by the production of IL-17, have also been shown to participate in the pathogenesis of PE. A predominantly Th17- and Th1-type response and decreased Treg immunity have been found in PE^25^. Excessive Th17 cell numbers and high levels of IL-17, IL-6, and IL-1β have been identified in PE^7^, indicating that uncontrolled Th17 cells may emerge as important mediators of inflammation and tissue damage in diseases of pregnancy. IL-27 counters the polarization of naive CD4^+^ T cells, resulting in the inhibition of Th17 cell development. IL-27 creates a high variety of biological activities that initiate and perpetuate an inflammatory response. Yin *et al.* observed that the expression of IL-27 and IL-27 receptor *α* were observely upregulation in trophoblastic cells from the placenta of PE compared with control subjects^9^.

The functional polymorphisms of cytokine genes was considered important for susceptibility, severity, and outcome of PE, therefore great attention has been focused on the role that these polymorphisms may play in the development of the diseases^11^,^26^,^27^. The production of cytokines is regulated by homologous genes, then these SNPs may play a important roles in the development of PE. Genetics and epigenetic may be involved in determining the phenotypes of diseases, and further study is needed to better understand the effect of genes with allelic or genetic heterogeneity on disease risk. Different polymorphisms in *IL27* have been found in association with a number of disorders, such as SLE^28^ and RA^29^. Among the *IL27* polymorphisms identified, rs153109 has been reported to be associated with the risk of asthma^30^, chronic obstructive pulmonary disease^31^, and inflammatory bowel diseases^32^, although it has no association with immune thrombocytopenia^33^.

In this study, we examined the genetic contribution of three polymorphisms in *IL27*, including rs153109 (5′ UTR region), rs17855750 (Exon 2/missense), and rs181206 (Exon 4/missense). To our knowledge, this is the first report investigation of the relationship between *IL27* polymorphisms and susceptibility to PE. Our results indicate that the frequency of the CC genotype of rs153109 is associated with a higher risk of presenting PE. The pregnant women bearing the CC genotype showed a 1.54-fold risk of developing PE, while the CT genotype was found to have a protective effect on PE. The distributions of genotypes and alleles of rs17855750 and rs181206 showed no difference in cases and controls. In conclusion, our study indicates that polymorphisms IL-27 may be involved in the development of PE in a Chinese Han population. Additionally, several studies have indicated that many candidate cytokine genes are thought to be associated with susceptibility to PE. Zubor *et al.* reported that the TNF-α G308A polymorphism was significantly associated with PE^34^, and IFN-γ (+874A) and IL-1β (-31C/T and -511T/C) are also associated with the risk of PE^11^,^14^. Besides, the little different between our data and data from HapMap may because that our current cases come from central China, while the populations examined by HapMap were from nouthern China. It is well known that various ethnic and environmental factors influence the analysis of LD plots and PE susceptibility. Therefore, we are unable to make comparisons between cohorts.

During the development and disease progression of PE, the basic pathological changes of systemic small vessel spasm often induce multi-organ disturbances, such as activation of the clotting system and impaired liver and renal function^35^. Our study showed that the serum levels of ALT, AST, urea nitrogen, and creatinine were higher in the PE patients compared with the controls, one reason may be that impaired liver and renal functions are common in PE patients. In our study, PE patients usually had an earlier gestational age at delivery and lower birth weight of offspring, leading to premature delivery.

This study has some inherent limitations that should be considered when interpreting our findings. First, there were a small number of patients diagnosed with partial or full HELLP syndrome or other severity of PE in this study. We didn’t distinct them due to severity of PE in small size, but this could add to the possible contribution of the SNP studied to the severity of the disease. Second, we only assessed three SNPs of *IL27*. The results obtained by this study may not completely represent the association between these SNPs and PE risk, and therefore the examination of more loci is needed to verify the association between *IL27* and PE. Third, because of the significant ethnic differences and population heterogeneity of PE patients worldwide, our study may not represent other ethnic groups. It remains important to determine whether *IL27* SNPs are associated with PE in multiple populations. Further functional analyses also need to be performed to clarify the potential mechanisms underlying the linkage between the rs153109 SNP and susceptibility to PE.

## Methods

### Study population

This study was approved by the Ethics Committee of the Affiliated Hospital of Qingdao University. Additionally, informed consent was obtained from all individuals after a thorough explanation of the procedure and its risk in accordance with the principles of the Declaration of Helsinki. PE patients and normal pregnant women (controls) were enrolled from the same geographic location, which included 1040 PE patients and 1247 controls from the Affiliated Hospital of Qingdao University, Linyi People’s Hospital and Heze Municipal Hospital, Binzhou Medical University Hospital, Yantai Yuhuangding Hospital, Yantaishan Hospital, Liaocheng People’s Hospital, and the Maternal and Child Health Care of Zaozhuang. The PE diagnosis was based on the criteria from the Report of the ‘National High Blood Pressure Education Program’^36^. Exclusion criteria included multiple pregnancies, fetal death, chronic hypertension and renal disease, uterine malformation, *in vitro* fertilization treatment, placental abruption, infection, cancer, gestational diabetes mellitus, or any other systemic disease, including pre-existing hypertension, systemic lupus erythematosus (SLE), and rheumatoid arthritis (RA). The controls were in the third trimester of normal pregnancy and had to be normotensive in the index pregnancy, without any fetal disorder, multiple pregnancy, or pathological states. Moreover, the controls were followed up and none of them developed PE after birth.

The research staffs filled out the questionnaire to all study objects, that contained maternal and gestational age, gravidity, the number of abortions, detailed medical, pregnancy and family history, systemic physical (including blood pressure) and pelvic examinations, complete blood counts, urinary analysis, blood clotting state, and routine biochemical.

### Genotyping

Genomic DNA was extracted from 2 mL ethylenediaminetetraacetic acid anti-coagulated peripheral blood samples using Qiagen DNA extraction kits (Qiagen, Shanghai, China). A spectrophotometer was used to test the concentration and quality of DNA. The purified DNA was stored at −80 °C until use. SNP genotyping was conducted using the TaqMan allelic discrimination real-time PCR method. The amplification primers used were as follows: rs153109 locus: forward 5′-TCGGGGCTCAGCCTGTGGCCAGGCT-3′, reverse 5′-GAGTTGAGTGAGGTCAGGATCAGGG-3′, rs17855750 locus: forward 5′-CCTGCATCTCGCCAGGAAGCTGCTC-3′, reverse 5′-CCGAGGTTCGGGGCCAGGCCCACCG-3′, rs181206 locus: forward 5′-ACCACGCTTCAGCCCTTCCATGCCC-3′, reverse 5′-GCTGGGAGGGCTGGGGACCCAGGGC-3′, which were designed and synthesized by Applied Biosystems by Life Technologies. Real-time PCR was performed on a LightCycler^®^ 96 System (Roche Diagnostics GmbH, Germany), and the amplifications were carried out with the following protocol: 95 °C for 3 min, followed by 45 cycles of 95 °C for 15 s and 60 °C for 1 min. For each cycle, the fluorescent signal from the VIC- or FAM-labeled probe was determined.

### Statistical analysis

Each SNP was assessed in the control group for departures from Hardy–Weinberg equilibrium using the Chi-squared (χ^2^) test. The χ^2^ test was used to compare the allelic and genotypic frequencies between the case and control groups using PLINK v1.07 (http://pngu.mgh.harvard.edu/Bpurcell/plink/). The analysis of variance (ANOVA) was used to analyze the laboratory examination of PE among different genotypes. The Pearson’s χ^2^ or Student’s t-test was used to test the comparisons of clinical data between groups. A *p*-value < 0.05 was considered statistically significant, and OR were calculated with exact CI of 95%. Three logistic regression models (additive, dominant, and recessive) were used to analyze the SNPs.

## Additional Information

**How to cite this article**: Liu, B. *et al.* Polymorphisms of the *IL27* gene in a Chinese Han population complicated with pre-eclampsia. *Sci. Rep.* **6**, 23029; doi: 10.1038/srep23029 (2016).

## Figures and Tables

**Table 1:** The clinical characteristics of cases and controls.

Characteristic	Cases (n = 1040)	Controls (n = 1247)	t-value	P-value
Age (years)	30.06 ± 5.69	30.32 ± 4.10	1.228	0.219
Gestational age (weeks)	35.48 ± 3.59	39.06 ± 1.52	29.176	<0.001
Neonatal gestational age (weeks)	36.29 ± 3.07	39.35 ± 1.30	28.262	<0.001
Birth weight of offspring (g)	2609 ± 916	3404 ± 370	24.628	<0.001
Age of menarche (years)	14.01 ± 1.22	14.09 ± 1.27	1.534	0.125
Systolic blood pressure (mmHg)	158.75 ± 18.98	113.93 ± 10.67	−67.246	<0.001
Diastolic blood pressure (mmHg)	103.75 ± 13.82	73.49 ± 7.83	−62.208	<0.001
Alanine aminotransferase (IU l^−1^)	23.92 ± 40.06	14.87 ± 14.67	−6.591	<0.001
Aspartate aminotransferase (IU l^−1^)	29.36 ± 45.68	18.88 ± 12.89	−6.876	<0.001
Urea nitrogen (mmol l^−1^)	5.25 ± 10.21	3.59 ± 10.30	−3.365	0.001
Creatinine (μmol l^−1^)	67.28 ± 26.60	54.89 ± 20.08	−11.057	<0.001

**Table 2:** Allele and genotype distribution of the IL27 gene markers in PE patients and healthy controls.

SNP	Allelic test					Genotypic test			
	Allele	Case/control	p	χ^2^	OR(95% CI)	Genotype	Case/control	p	χ^2^
rs153109	C	827/938	0.14	2.23	1.10(0.97–1.24)	CC	198/165	7.83 × 10^−5^	19.06
	T	1242/1542				CT	431/608		
						TT	405/467		
rs17855750	G	278/359	0.29	1.14	0.91(0.77–1.08)	GG	27/34	0.53	1.29
	T	1750/2061				GT	224/291		
						TT	763/885		
rs181206	C	297/345	0.59	0.29	1.05(0.86–1.24)	CC	27/32	0.84	0.35
	T	1725/2099				CT	243/281		
						TT	741/909		

**Table 3:** Analysis of the three SNPs based on three genetic models.

SNP	Additive model		Dominant model		Recessive model	
	p	OR(95%CI)	p	OR(95%CI)	p	OR(95%CI)
rs153109	0.14	1.09(0.97–1.23)	0.46	0.94(0.79–1.11)	1.63 × 10^−4^	1.54(1.23–1.93)
rs17855750	0.26	0.90(0.74–1.08)	0.30	0.92(0.78–1.08)	0.83	0.94(0.57–1.58)
rs181206	0.56	1.05(0.89–1.23)	0.56	1.28(0.88–1.28)	0.94	1.02(0.61–1.72)

**Table 4:** Associations between genotypes of rs153109 and characteristics among PE patients.

db SNP ID (allele 1/allele 2)	(1) CC	(2) CT	(3) TT	(1) vs. (2) vs. (3), *P*	(1) vs. (2) vs. (3), *P*_*C*_	(1) vs. (2), *P*	(1) vs. (2), *P*_*C*_	(1) vs. (3), *P*	(1) vs. (3), *P*_*C*_	(2) vs. (3), *P*	(2) vs. (3), *P*_*C*_
Age (years)	30.16 ± 5.45	30.14 ± 5.77	29.92 ± 5.7	0.81	–	0.98	–	0.63		0.56	–
Gestational age (weeks)	35.65 ± 3.43	35.04 ± 3.66	35.81 ± 3.48	**0.006**	**0.018**	0.05	0.15	0.59	–	**0.002**	**0.006**
Neonatal gestational age (weeks)	36.41 ± 3.00	35.91 ± 3.19	36.61 ± 2.93	**0.006**	**0.018**	0.08	0.24	0.46	–	**0.002**	**0.006**
Birth weight of offspring (g)	2644 ± 917	2507 ± 915	2686 ± 906	**0.02**	0.06	0.10	0.3	0.63	–	**0.008**	**0.024**
Age of menarche (years)	13.88 ± 1.18	14.08 ± 1.18	14.00 ± 1.27	0.17	0.51	0.06	0.18	0.24	0.72	0.39	–
Systolic blood pressure (mmHg)	157.63 ± 18.27	159.93 ± 20.19	158.19 ± 17.98	0.26	0.78	0.16	0.48	0.73	–	0.19	0.57
Diastolic blood pressure (mmHg)	103.04 ± 12.8	104.70 ± 14.15	103.10 ± 13.94	0.18	0.54	0.17	0.51	0.96	–	0.10	0.3
Alanine aminotransferase (IU l^−1^)	23.26 ± 27.95	24.01 ± 31.93	23.69 ± 51.04	0.98	–	0.83	–	0.91	–	0.91	–
Aspartate aminotransferase (IU l^−1^)	29.87 ± 39.35	29.38 ± 33.48	28.23 ± 57.80	0.90	–	0.91	–	0.69	–	0.72	–
Urea nitrogen (mmol l^−1^)	4.60 ± 1.97	5.00 ± 2.97	4.90 ± 3.59	0.34	–	0.14	0.42	0.27	0.81	0.66	–
Creatinine (μmol l^−1^)	64.81 ± 21.56	67.97 ± 23.34	67.84 ± 31.82	0.36	–	0.18	0.54	0.21	0.63	0.95	–

*P*_*C*_ *P* value corrected by Bonferroni method.
